# rTMS for treatment resistant depression – a single center community experience

**DOI:** 10.3389/fpsyt.2026.1892766

**Published:** 2026-08-05

**Authors:** Francois Henri Jacques, Camille Godin, Nassima Rabia, Bradley Erik Apedaile

**Affiliations:** 1 Clinic Neuro-Outaouais Gatineau, Gatineau, QC, Canada; 2 School of Psychology, Faculty of Social Sciences, University of Ottawa, Ottawa, ON, Canada

**Keywords:** antidepressant, depression, major depression disorder, rTMS, treatment resistant depression

## Abstract

Depression remains a major health issue worldwide. Chronic treatment resistant depression, where there is a failure to respond to two or more antidepressant regimens, affects 2.5 percent of the adult population. Repetitive transcranial magnetic stimulation (rTMS) is an accepted treatment modality for depression. The comprehension of its mechanism of action and optimal parameters for treatment are evolving. We performed a chart review of the patient population with treatment resistant depression treated at our center with rTMS over the past ten years, using a sequential bilateral hemisphere stimulation approach. Patients were evaluated using the Beck Depression Inventory questionnaire (BDI-II) before onset and every 2 weeks during their rTMS treatments. We also attempted to contact the patients who had completed treatment to repeat the BDI-II and assess long term impacts of treatment. Fifty-three patient charts were analyzed. The overall incidence of responders and those who achieved remission was 58.5% (n=31) and 20.8% (n=11) respectively. Nine patients were contacted following completion of treatment and completed the BDI-II evaluations. The average time between the completion of their rTMS treatment and follow-up was 2.6 years (SD = 2.7 years). Eight of the nine had achieved a significant improvement during their rTMS treatment and 6 maintained their benefit. Overall, our results suggest that using a sequential bilateral hemisphere rTMS stimulation approach delivered in a real-world community for unselected patients with chronic, severe, treatment resistant depression is effective with efficacy results comparable to those achieved in clinical trials of major depression and that in some cases, the clinical benefit can persist over the long term.

## 1 Introduction

From a global perspective, major depressive disorder (MDD) is one of the top three disorders associated with years lived with disability (YLD), accounting for 5.7 percent of all causes. Its recent prominence is the result of a 49.7 percent increase between 2010 and 2023 (1). The US adult lifetime prevalence of MDD was estimated in 2018 at 20.6 percent (2). MDD is often a recurrent or chronic condition with relapses occurring after the first depressive episode in up to 75 percent of cases (3), and about 30 percent of depression becomes persistent (4). The lifetime prevalence of recurrent depression was estimated to be 10.5 percent, while persistent depression was between 1.6–18 percent (5). Adequate medical management of MDD remains an unmet need. The STAR*D trial (Sequenced Treatment Alternatives to Relieve Depression) showed a cumulative remission rate following multistep medical treatment of 67 percent with remission rates tending to diminish with each step from 36.8 percent following the first intervention to 13.7 percent after the third intervention, accompanied by an increasing risk of relapse (6).

Treatment resistant depression (TRD) is a form of depression where there is a failure to respond to two or more antidepressant regimens despite adequate dose, duration and adherence to treatment (6–8). Approximately 2.5 percent of the population live with chronic TRD (9). For patients with MDD and especially TRD, repetitive transcranial magnetic stimulation (rTMS) represents a potentially effective alternative therapeutic modality.

Management of TRD has moved beyond conventional antidepressant therapies and now encompasses a range of augmentation therapies and rapid acting strategies. Augmentation therapies, including lithium and atypical antipsychotics, along with dose optimization and class switching strategies, are used for patients who partially respond to conventional treatments. More recently, there is evidence that glutamatergic dysfunction contributes to the pathogenesis of TRD, driving interest in N-methyl-D-asparate (NMDA) receptor antagonists. Esketamine in a nasal spray was approved in 2019 by the FDA for treatment of TRD and a growing body of clinical research has validated its efficacy, tolerability and safety. In parallel, psychedelic-assisted therapies, notably psilocybin along with other serotonergic agents have shown promise in the treatment of a variety of psychological disorders, including mood disorders, although their use remains largely experimental (10). These novel interventions have been compared to accelerated neurostimulation protocols. Pettorruso et al., for example, found that accelerated high frequency rTMS resulted in a faster antidepressant response than intranasal esketamine at 1 month, with a comparable response rate by three months and a lower incidence of treatment related side effects (11). Nevertheless, within this evolving therapeutic landscape, rTMS remains a distinct, non-invasive, and well tolerated option for treatment of MDD and TRD.

rTMS belongs to a broader, evolving family of non-invasive brain stimulation (NIBS) approaches for TRD. Transcranial direct current stimulation (tDCS) is another NIBS modality that has been investigated as an alternative treatment for TRD. In contrast to rTMS, which generates, transient focal currents through electromagnetic induction, tDCS delivers a weak, continuous direct electrical current through electrodes placed directly on the scalp. As with rTMS, the dorsolateral prefrontal cortex is typically the target in tDCS trials for depression. A recent review by Moshfeghinia et al. (12) concluded that tDCS may offer only modest, short-term benefits in TRD, with insufficient evidence for durable antidepressant effects. Compared to tDCS, a recent consensus review of rTMS in MDD (13) reiterated the strong evidence for the short-term effectiveness of rTMS with reported response rates of 58 to 83 percent, and remission rates of 28 to 62 percent (14). However no relevant baseline demographic characteristic has shown a reproducible predictive value (13, 15–17).

The durability of the antidepressant effect of rTMS in MDD remains understudied. A meta-analysis reported sustained response rates of 66.5 percent, 52.9 percent and 46.3 percent at 3, 6 and 12 months respectively (4). However, the review included studies that incorporated maintenance treatment protocols. Notably, six-month sustained response rates were higher in studies using maintenance treatment (61.1%) compared to those without maintenance protocols (38.5%) (4). These findings suggest that maintenance treatment may play an important role in sustaining clinical benefits, and although a few clinical trials have studied various maintenance treatment protocols (18–21) the optimal form and duration of maintenance rTMS treatments remains to be determined.

### 1.1 Neurobiological roots of MDD

Depression is the result of the interactions between neurobiological, environmental and psychosocial factors (22). Neurobiological factors include hypofunction and atrophy of the dorsolateral prefrontal cortex and hippocampus, a reduction in hippocampal neurogenesis-related events and dendritic density (23–26). Meneses-San Juan et al. demonstrated in animal models how rTMS can reverse these structural changes and promote formation of dendritic spines, synaptic connections in the frontal cortex and dentate gyrus through epigenetic mechanisms (27). Quan et al. have shown that rTMS can promote synaptic plasticity and connectivity and increase transcription of neurotrophic factors such as BDNF, TrkB, and NMDA receptors (28).

Computational neuroscience proposes the attractor theory of depression (29). An attractor network is a network of neurons that feedback onto itself through associative, modifiable, and recurrent collateral synaptic connections. It is prototypical of the neocortex and hippocampus and forms the physiological basis for long- and short-term memory and the top-down bias in attention and decision making. The theory proposes that depression arises from an overly sensitive and hyperactive non-reward punishment attractor network centered in the lateral orbitofrontal cortex. Overly sensitive networks may emerge through genetic predisposition and/or through prolonged exposure to sustained negative experiences. Increased functional connectivity between this network and other cortical areas involved in self-awareness (precuneus), language (angular gyrus) and attention (dorsolateral prefrontal cortex) may contribute to the biological basis for the clinical symptoms of depression. These areas may in turn provide recurrent feedback to the lateral orbitofrontal cortex, further reinforcing the stability and persistence of the negative mood-related attractor state. This process may be compounded by a reduced sensitivity, and underactive and decreased functional connectivity within the medial orbitofrontal reward network, particularly in its connections to the parahippocampal cortex, anterior cingulate gyrus, and temporal cortex. These alterations may also contribute to the decreased stability and persistence of positive mood attractor states (29).

Inflammation is intimately associated with depression (30–34) and the role of oxidative stress (OS) in the initiation and maintenance of depression is well established (35). The brain is particularly vulnerable to reactive oxygen species (ROS) as it has an intrinsically low antioxidant capacity which is mediated by astrocytes and a high rate of oxygen consumption and high unsaturated lipid content which predisposes to lipid peroxidation. Monoamine oxidase activity is influenced by ROS, which in turn can induce ROS in mitochondria. The accumulation of ROS within the brain can promote inflammation and activate the inflammasome within microglia, further contributing to the symptomatology (36).

At the molecular level, under conditions of chronic microglial and astrocyte activation, inducible nitric oxide synthase (iNOS) is upregulated, generating excess NO that reacts with superoxide to form peroxynitrite, a highly reactive oxidant responsible for DNA damage, nitration of tyrosine residues on neuronal proteins and direct impairment of mitochondrial electron complexes I and III, ultimately promoting neuronal apoptosis (37). This cascade of oxidative and nitrosative stress is damaging to hippocampal and prefrontal circuits that cover mood regulation and cognitive function with declining antioxidant enzyme activity which amplifies neuronal vulnerability to ROS. Mitochondrial ROS accumulation further activates the NLRP3 inflammasome, triggering the release of pro-inflammatory cytokines which contributes to the persistence of symptoms in TRD (37). rTMS may provide antidepressant effects in part by restoring this redox equilibrium. Tian et al. showed that rTMS can improve depression in animal models by enhancing the anti-inflammatory effect of the Nrf2 signaling pathway (38, 39). rTMS has also been shown to induce the polarization of immune cells such as astrocytes (40) and microglia (41, 42) and shift cytokine production to a non-inflammatory profile (43, 44).

Further, depression is classically associated with a reduction in several neurotransmitters including serotonin, dopamine, gamma-aminobutyric acid (GABA) and glutamate (45, 46). rTMS has been shown to increase neurotransmitter (dopamine, serotonin, glutamate, GABA, acetylcholine) levels in the frontal, striatal and limbic areas (47, 48).

### 1.2 Potential mechanisms behind rTMS efficacy

rTMS can modulate membrane excitability of both neurons and inhibitory interneurons with the change in the balance between the two possibly contributing to its clinical benefit (49). Functional MRI studies have demonstrated how rTMS induces changes in functional connectivity within the frontostriatal network, and between the dorsolateral prefrontal cortex (DLPFC) and default mode network and how these may potentially underlie its clinical benefit. Abnormalities in functional connectivity have also been shown to distinguish between MDD and bipolar disorder and identify potential responders to rTMS (50–53).

High frequency (10 Hz) unilateral stimulation to the left DLPFC remains the most widely studied and used rTMS stimulation protocol for MDD. Other protocols using the same or different coils and targeting techniques continue to be investigated. There is a school of thought proposing a linear dose response model whereby a greater number of sessions, higher stimulation intensity, and a greater number of stimulations per session correlate with greater efficacy (13, 54). Recent evidence suggests a different perspective. Yu et al, through a metanalysis of dose response in TRD patients found no further clinical gain following a total cumulative dose of 26,660 pulses (55). Oostra et al. and Tang et al. (56, 57) found instead a linear gain with increasing number of sessions with an approximate optimal number of pulses per session of 1200-1500. A recent SHAM -controlled meta-analysis comprising 1850 patients with MDD reiterated the robust anti-depressant effect of rTMS with a number need to treat of 2.9 for response and 4.4 for remission. The meta-analysis was also unable to identify any stimulation parameter that exerted a linear dose response influence on the antidepressant effect. The author proposed that rTMS efficacy is more likely to be constrained by a biological optimum rather than a dose dependency (16). One of the unanswered questions pertains to whether using a bilateral, sequential, high frequency stimulation to the left (DLPFC) and low frequency over the right DLPFC is superior in efficacy to the unilateral stimulation of only the left DLPFC (13, 30, 58). Fitzgerald et al. (59) showed that sequential bilateral rTMS was superior to SHAM with a 44% response, 36% remission rate compared to 8% response and 0% remission rate for the placebo group. Trevizol et al. found that bilateral rTMS resulted in a 40% remission rate compared to 0% for either unilateral or SHAM rTMS (60), while Blumberger et al. showed that bilateral stimulation was superior to SHAM while unilateral stimulation was not (58). In terms of stimulation frequency, theta burst stimulation protocols have gained in popularity because of its stimulation time, but Wada et al. (61) were unable to show a non-inferiority of bilateral intermittent theta burst versus bilateral rTMS.

In this study, we sought to characterize real-world efficacy of rTMS for TRD in a clinically heterogeneous, unselected patient sample treated within a community setting in Canada, comparing our outcomes with published efficacy data for MDD, and assessing the durability of treatment benefits through long-term follow-up.

## 2 Materials and methods

### 2.1 Sample

We did a retrospective chart review of patients who received rTMS for TRD at our clinic in Gatineau, Canada between 2015 and 2025. Demographic and efficacy data were collected and analyzed. We attempted to contact each patient post treatment between 2024 -2025, to repeat the Beck Depression Inventory (BDI) questionnaire.

All the patients in this review were referred to our clinic by their primary care physician or psychiatrist with a diagnosis of major depressive disorder having failed multiple conventional treatment modalities. Other than their diagnosis, we did not have access to details on whom made the diagnosis, how prior treatment adequacy was established and on comorbid psychiatric conditions. In this retrospective chart review, these details were not consistently available.

Ethical approval was granted by the Canadian SHIELD Ethics Review Board (approved October 23, 2023; Principal Investigator: Dr. Jacques). A note to file was received by Canadian Shield for patients who we were unable to contact and who had received their treatments prior to 2023 agreeing to the use of the anonymized data in our research.

### 2.2 Equipment and stimulation procedure

rTMS treatments were delivered using a neuro-navigation-guided stimulation system (Rogue Research, Montreal Canada) to localize cortical targets and ensure consistent coil positioning and orientation throughout treatment sessions. Resting motor threshold (RMT) was determined using a Nicolet Viking EDX electromyography (EMG) system (Natus Medical Incorporated, Middleton USA). During motor threshold determination, patients were in the supine position and provided with ear plugs for protection. Using the neuro-navigation system, a butterfly coil (D70A) was positioned over the hand area of the primary motor cortex (M1), and the RMT was established based on motor-evoked responses recorded with the EMG system. Following threshold determination, the D70A coil was repositioned over the target left dorsolateral prefrontal cortex (DLPFC), and either the sequential bilateral stimulation protocol or theta burst stimulation protocol was initiated.

### 2.3 Treatment protocol

Patients receiving rTMS for TRD at our clinic routinely underwent sequential bilateral stimulation of the DLPFC with a stimulation frequency of 1 Hz over the right hemisphere and 10 Hz over the left for a total of 2700 stimulations (1500 on the right and 1200 to the left DLPFC) per session. Eight patients were treated using a theta burst protocol which included the right and left DLPFC and a third site over the Pz area for a total of 1800 stimulations (600 per site) per session. Patients were instructed to continue their antidepressant medication during the rTMS treatment and were encouraged to complete 30 sessions, typically administered once per day (Monday to Friday) over 6 weeks. Evaluations using the Beck Depression Inventory II (BDI-II) was carried out at baseline (Timepoint 0) and repeated after every 10 sessions (Q2 weeks) for a total of 4 evaluations (Timepoints 0 to 3). Patients who received 40 sessions had a 5^th^ evaluation at timepoint 4. Details of the rTMS methods can be found in the supplementary material.

### 2.4 Clinical assessment

Patients were assessed at baseline and after each 2-week treatment course (approximately 10 treatments) using the Beck Depression Inventory II (BDI). The BDI-II is a widely used 21-item self-administered questionnaire measuring the severity of depressive symptoms in adults and adolescents but is not a diagnosis tool. Each item is scored on a 4-point scale ranging from 0 to 3, yielding a total score between 0 and 63, with higher scores indicating greater severity and scores of 10 or less considered as normal or not clinically depressed. The instrument shows strong psychometric properties including high sensitivity, reliability and structural validity (62). It assesses symptoms across both cognitive-affective and somative-vegetative dimensions, capturing symptoms such as sadness, pessimism, guilt, self-criticism, suicidal ideation, changes in sleep, appetite, energy and sexual interest (63).

### 2.5 Statistical analysis

All statistical analysis was performed using R version 4.6.0 (64). Descriptive statistics were used to characterize patient demographics. We used a linear mixed effects model using the lmerTest package, version 3.2–1 to model BDI-II scores at each timepoint compared to baseline (65). Sex, age and treatment type were also added to the mixed effects model to explore their predictive value to the BDI outcome. T-tests were used to compare the BDI scores of response versus non-response to treatment and remission versus response at each timepoint. The primary efficacy endpoint was the final observed BDI-II score following treatment, excluding the scores collected at long-term followup. Response was defined as a greater than 50% reduction in the BDI-II score from baseline to the final observed BDI-II score and remission was defined as a BDI-II score 10 or less at the final observed BDI-II score.

## 3 Results

A total of 53 patient charts were reviewed. The mean age of patients was 41.1 years (19 – 72) and 41.5 percent (n = 22) male. All patients suffered from chronic, treatment resistant depression that had been ongoing for several years before being referred to our clinic by their primary care physician or psychiatrist. Most were no longer working or attending school, and all had failed multiple (more than 3) antidepressant medications. The BDI-II score of males at baseline were not significantly different from those of females at baseline (p = 0.51). Females were significantly younger than males at baseline (37.4 years vs 46.3 years, p = 0.03). Age, sex and treatment type (bi-hemispheric versus theta burst) were not significant predictors of BDI-II scores in the linear mixed effects model.

The patients in our review were clinically heterogeneous. In addition to TRD, participants presented with a range of comorbid psychiatric diagnoses, including post-traumatic stress disorder (8 patients) as well as other conditions such as, attention deficit/hyperactivity disorder, personality disorders, and substance use disorders (4 patients). By not excluding patients based on psychiatric comorbidities, this study captures a heterogeneous, unselected clinical population and reflects a real-world rTMS treatment setting outside of a highly controlled clinical trial or specialized clinic.

Forty of the 53 patients (75.5%) completed between 20 and 34 rTMS sessions (average number of session = 29.3), over the standard 6-week treatment course used at our clinic. Ten patients (18.9%) underwent an extended treatment protocol of an additional 4 to 17 treatments (mean number of additional treatments = 9.6) over an additional 2-week treatment course, typically due to partial but ongoing clinical improvement. Thirteen patients discontinued treatment early, 10 after 20 treatments over 4 weeks of treatment and 3 after less than 20 treatments. Of those thirteen patients, 8 had responded to treatment, of which 5 were in remission at the time they discontinued treatment. Five patients discontinued treatment after having had no response. Overall, patients received between 10 and 47 total treatments over 2 to 8 weeks. Most patients received approximately 30 treatments over 6 weeks. Four patients received bilateral intermittent theta burst stimulations (iTBS) and 4 patients received a combination of bi-hemispheric and iTBS stimulations.

**Figure 1:**
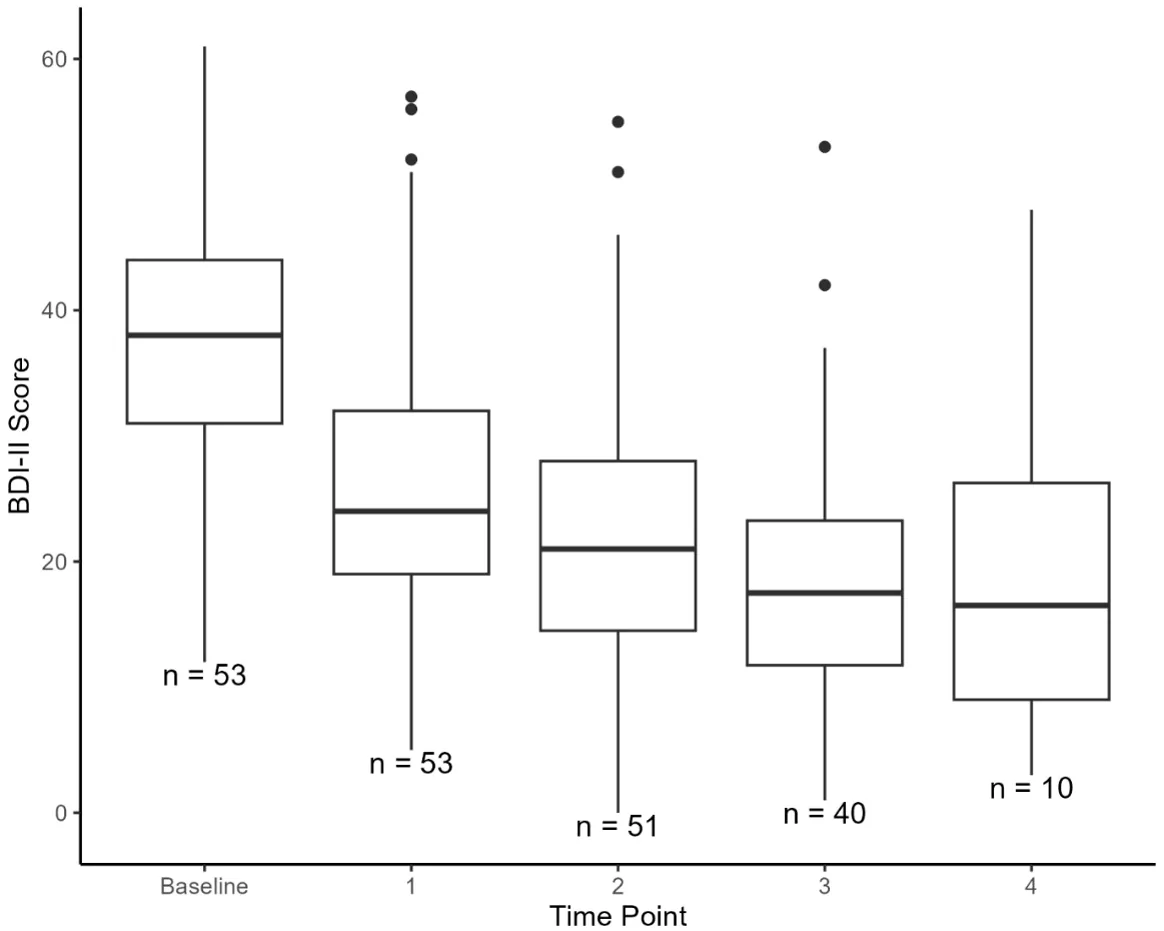
Overall BDI score by timepoint. Boxplots of Beck Depression Inventory II (BDI-II) scores across treatment time points. Boxes represent the interquartile range, the central line indicates the median, and whiskers represent score variability across participants.

Overall, the group mean BDI-II score decreased progressively starting with timepoint 1 (after 10 treatment sessions from baseline) achieving a 50 percent reduction from baseline to timepoint 3 after 30 treatment sessions (**Figure 1**). The results of the linear mixed effects model are presented in **Table 1**. The model with timepoint as the only fixed effect shows a significant decline in BDI-II score across all timepoints relative to baseline, with the magnitude of the incremental decline in between timepoints decreasing with the number of treatments. The intraclass correlation coefficient of 0.59 indicates that 59 percent of the total variance of BDI-II scores is attributed to between patient differences. A second model adding sex, age and treatment type (bi-hemispheric and theta burst) as fixed effects did not significantly improve the model fit. None of age, sex or treatment type was independently associated with BDI-II scores after accounting for time. Using t-tests to compare the change in the mean BDI-II scores between timepoints, we found that the drop in BDI-II between Baseline and Timepoint 1 and the drop in BDI-II between Timepoint 1 and Timepoint 2 were significant, while the change in BDI-II between Timepoint 2 and Timepoint 3 and between Timepoint 3 and Timepoint 4 were not significant. This suggests that the bulk of the benefit from the treatment is seen early on after 10 to 20 sessions and continues in smaller increments with subsequent treatments, and that more than 30 consecutive daily sessions did not appear to contribute significantly to the overall benefit from the rTMS therapy.

**Table 1:** Linear mixed effects model summary.

Basic model				
Predictor	Estimate (CI)	SE	df	p
Intercept	36.83 (33.6 – 40.0)	1.61	100.4	<0.001
Timepoint 1	-10.39 (-13.3 - -7.51)	1.45	150.4	<0.001
Timepoint 2	-15.10 (-18.0 - -12.2)	1.47	151.0	<0.001
Timepoint 3	-18.85 (-22.0 - -15.7)	1.60	153.1	<0.001
Timepoint 4	-20.72 (-26.2 - -15.3)	2.77	157.4	<0.001
Extended model				
Intercept	34.43 (25.6 – 43.3)	4.43	54.9	<0.001
Timepoint 1	-10.53 (-13.4 - -7.6)	1.46	148.3	<0.001
Timepoint 2	-15.24 (-18.2 - -12.3)	1.48	149.0	<0.001
Timepoint 3	-19.02 (-22.7 - -15.8)	1.62	151.0	<0.001
Timepoint 4	-20.54 (-26.0 - -15.0)	2.79	156.4	<0.001
Age	0.06 (-0.15 – 0.28)	0.11	50.3	0.55
Sex	-0.48 (-6.30 – 5.34)	2.90	49.1	0.87
Tx Type BH	7.72 (-2.64 – 18.11)	5.25	167.3	0.14
Tx Type TB	-0.25 (-5.81 – 5.30)	2.82	191.8	0.93

### 3.1 Individual clinically significant response

The primary endpoint was the change in BDI score from baseline to the final treatment session. Patients were divided into responders, non-responders, and those whose achieved remission. Response was defined as a reduction from baseline to the last treatment session in the BDI-II score of 50 percent or more while remission was defined as a reduction in BDI-II score 10 or less at the last treatment session.

**Figure 2:**
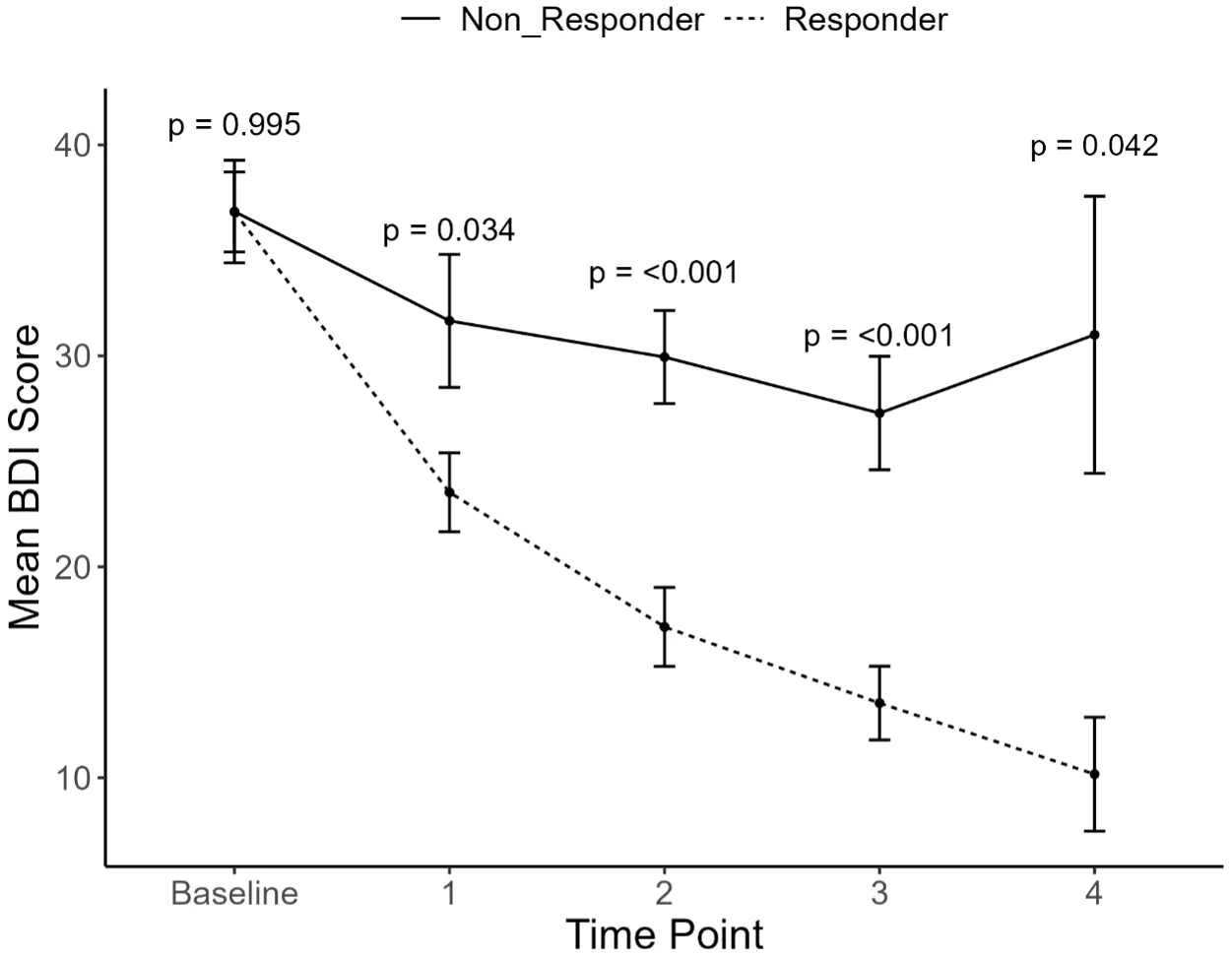
Mean bdi score responders vs non-responders. Mean Beck Depression Inventory (BDI) scores across treatment time points for clinically significant responders (dashed line) and non-responders (solid line). Clinically significant response was defined as a reduction of at least 50% from baseline BDI score. Error bars represent standard error of the mean. .

**Figure 2** shows, as would be expected, that responders had significantly lower BDI scores across all Timepoints compared to non-responders and that while responders show improvement at each timepoint, non-responders show relatively little change in BDI-II over time.

The overall incidence of responders and those who achieved remission was 58.5% (n=31) and 20.8% (n=11) respectively (**Table 2**). Age, sex, and baseline BDI-II scores did not predict response to rTMS in our cohort of TRD patients. The highest absolute number of patients achieving a response was at Timepoint 3 (55% n=22) and those with remission at Timepoint 2 (18%, n=10). Patients who did not improve significantly by Timepoint 2 (4 weeks) tended to not respond to additional treatments (**Figure 2**).

**Table 2:** Demographics and BDI of non-responders, responders and remissions.

Variable		Non-responder	Responder	Remission
	n (%)	22 (41.5)	31 (58.5)	11 (20.8)
Age (mean)	Mean	41.3	40.9	40.3
Sex n (% Female)	n (% Female)			
BDI-II Baseline	Mean	34.4	38.6	33.1
BDI-II Final	Mean	29.1	11.8	5.5
Change in BDI-II	%	-15.4	-69.4	-83.3

Patients in remission are a subset of the responders.

### 3.2 Durability of response

We were able to contact 9 patients out of the original 53 to repeat BDI-II evaluations. Patients were contacted on average 2.6 years after the last treatment (SD = 2.7 years; median = 1 year), with a minimum interval of 3 months and a maximum interval of 9 years. **Table 3** provides a comparison of follow-up and non-follow-up patients. The follow-up and non-follow-up patients were similar in terms of age, sex and number of treatments.

**Table 3:** Comparison of follow-up and non-follow-up patients.

Variable	n (%)	Follow-up	Non-follow-up	p value1
		9 (17)	44 (83)	
Male	n (%)	3 (33)	19 (43)	0.60
Age	mean (sd)	39.4 (13.6)	41.5 (13.9)	0.68
No of Treatments	mean (sd)	34.4 (6.2)	27.4 (6.5)	0.13
1p values calculated using Welche’s two sample t-tests				

At the study endpoint, 6 of the 9 patients in the follow-up group were in remission, 2 were responders and 1 was a non-responder. At follow-up, 3 patients remained in remission, 1 remained in response, 2 moved from remission to response, 2 moved from remission to non-response, and the non-responder remained a non-responder.

## 4 Discussion

In this study, we performed a retrospective chart review of a clinically heterogeneous group of patients who underwent rTMS treatment for TRD. rTMS though effective has not yet become widely available in Canada for treatment of depression. There are as yet no large studies specifically looking at treatment of patients with TRD using rTMS. Nevertheless, rTMS is emerging as a promising intervention for treatment-resistant depression, although its efficacy in routine clinical practice and in more heterogeneous patient populations remains understudied. Our study shows that, in an unselected real-world sample of unselected patients with chronic, severe TRD, often complicated by psychiatric comorbidities, a bilateral stimulation protocol produced antidepressant effects comparable to those reported in clinical trials of MDD. Our results are consistent with those reported by Trevizol et al. (60), Blumberger et al. (30) and Fitzgerald et al. (59), suggesting that a bilateral sequential stimulation protocol is effective. Whether a bilateral approach offers greater or similar therapeutic benefits to unilateral stimulation protocols remain to be determined by a large head-to-head study. However, we can speculate that a sequential bilateral stimulation protocol may take greater advantage of the purported mechanisms of action of rTMS. A possible framework supporting the latter involves the attractor theory of depression (29) which proposes that the imbalance does not lie between the left and right dorsolateral prefrontal cortices, but rather between the lateral and medial orbitofrontal cortices in both hemispheres, with downstream alterations in functional connectivity involving regions such as the dorsolateral prefrontal cortex. From this perspective, a bilateral stimulation approach such as the one we used in our refractory chronic TRD population, may be better suited than the conventional unilateral left dorsolateral prefrontal cortex stimulation to restore the neurodynamic equilibrium between the lateral orbitofrontal non-reward/punishment networks and medial orbitofrontal reward-related network. Re-establishing this balance may help recalibrate the feedforward and feedback interactions between lateral orbitofrontal cortex with the dorsolateral prefrontal, angular, and precuneus cortices in both hemispheres, thereby alleviating depressive symptomatology. Our small follow-up cohort suggests that, in some patients, this re-established equilibrium may remain stable over time (mean follow-up: 2.5 years). Maintenance rTMS could potentially improve on the latter.

The clinical benefits observed in our TRD cohort are consistent with a growing body of evidence linking rTMS efficacy to the restoration of cerebral redox homeostasis, reduction in cerebral inflammation and improved functional connectivity.

As with most rTMS trials, we were unable to detect any baseline demographic predictive factor. The brunt of the benefit appears to occur early in the course of treatment, about 2 weeks after onset. Although non-responders demonstrated some symptom improvement, the magnitude of change was insufficient to reach clinical significance. As a group, their improvement tended to plateau after approximately 20 sessions (week 4), with negligible additional benefit observed following an extra 10 sessions.

## 5 Limitations

The retrospective and uncontrolled design of our study and the absence of a SHAM or comparison group makes it difficult to account for placebo effects, spontaneous symptom fluctuations, or the influence of concurrent treatments. The sample size was relatively small, particularly for the long-term follow-up cohort, which limits the generalizability of the results. In our sample, where patients were classified as non-responders according to our criteria, the heterogeneity of the cohort makes it difficult to explore the role that underlying psychological comorbidities play in non-response to rTMS therapy. Our study focused solely on resolving depressive symptoms and not on related comorbidities such as anxiety. It would be worthwhile to consider new protocols that could target multiple pathways simultaneously. Our clinically heterogeneous sample, although a fair representation of routine clinical practice, may also have introduced variations related to the comorbidities. We did not have access to data on comorbid conditions and did not control for them in our modeling. Furthermore, residual symptoms, which are lingering symptoms that persist after treatment, may be responsible for a high relapse rate and progression to chronic depression. Formal analysis of predictors of early discontinuation of treatment was not possible due to small sample size resulting in a lack of statistical power. Finally, it is important to note that the diagnosis of depression is based upon clinical evaluations. While psychometric scales are useful for measuring severity and monitoring progression, they remain measures based upon patients’ experiences, which are personal and subjective. These measures, such as the BDI-II which we used in our study, do not replace a complete medical evaluation of a depressive state.

## 6 Conclusions

Sequential bilateral DLPFC rTMS delivered in a real-world community setting appears to be an effective treatment for patients with chronic, severe TRD, with outcomes comparable to those reported in clinical trials of MDD. Although baseline demographic characteristics did not predict treatment response, clinical trajectories generally became distinguishable after approximately 20 treatment sessions (week 4). Our findings also suggest that, in some patients, the therapeutic benefits of rTMS may persist over the long term.

## Data Availability

The raw data supporting the conclusions of this article will be made available by the authors, without undue reservation.
