# Supplementary material for: rTMS for treatment resistant depression – a single center community experience
Source: Front Psychiatry. 2026 Aug 5;17:1892766. doi: 10.3389/fpsyt.2026.1892766 (PMC13487954; doi:10.3389/fpsyt.2026.1892766)
Supplement: Supplementary file 1 [file Table1.docx]

rTMS for treatment resistant depression - a single center community experience -Supplementary Material

1. Equipment

A Magstim Rapid 2 (Magstim Whitland,U.K.) with a D70A butterfly coil was used.

The electric and magnetic fields produced according to distance from the coil surface at 100% maximum machine output are shown in Table A-1 below.

Table A- 1 MagStim Rapid-2 TMS E-Field and Bx-Field at Maximum Output

| Distance from magnet surface | E field^1^ DCC^2^ | E field D70A^3^ | Bx^4^ field DCC | Bx field D70A |
| --- | --- | --- | --- | --- |
|  | (V/m) | (V/m) | (Tesla) | (Tesla) |
| 1cm | 331 | 175 | 0.65 | 0.65 |
| 2cm | 242 | 111 | 0.50 | 0.40 |
| 3cm | 174 | 71 | 0.39 | 0.26 |
| 4cm | 123 | 45 | 0.31 | 0.18 |
| 5cm | 84 | 29 | 0.24 | 0.13 |
| 6cm | 55 | 18 | 0.18 | 0.09 |
| 7cm | 35 | 11 | 0.15 | 0.07 |
| 8cm | 21 | 6 | 0.11 | 0.06 |
| ^1^ E field – Electric Field | | | | |
| ^2^DCC - Double Cone Coil | | | | |
| ^3^D70A – Butterfly Coil | | | | |
| ^4^Bx – Magnetic Field | | | | |

A neuro-navigation system (**Rogue Research**, Montreal, Canada) was used to identify the stimulation sites and to orient the coils.

A **Nicolet Viking EDX EMG** (**Natus,** Middleton, USA) machine was used to determine the resting motor threshold.

2. **Stimulation Procedure**

- Patients were provided ear plugs.
- While in a supine position using the neuro-navigation system the butterfly coil was placed over the hand M1 of hand and the resting motor threshold was determined using the Viking EMG Machine
- The Butterfly coil (D70A) was then placed over the target left DLPFC and the bilateral stimulation protocol or the theta burst protocol was initiated
  a. **Bilateral stimulation protocol**

| **Right DLPFC** | **Left DLPFC** |
| --- | --- |
| **Coil Target** Right DLPFC  **Frequency** 1 Hz  **Power** 110% of the Motor Threshold  **Train Duration** 10 seconds  **Pulses per Train** 10  **Inter-train Interval** 1 second  **Number of Trains** 150  **Total Pulses** 1500 | **Coil Target** Left DLPFC  **Frequency** 10 Hz  **Power** 110% of the Motor Threshold  **Train Duration** 3 seconds  **Pulses per Train** 30  **Inter-train Interval** 25 seconds  **Number of Trains** 40  **Total Pulses** 1200 |

b. **Theta burst stimulation protocol**

The intermittent Theta Burst Stimulation (iTBS) protocol consists of three stimulation targets applied within the same session: the right DLPFC, the left DLPFC, and an interhemispheric midline target (Pz) using the following parameters.

- **Power** 65% of maximum machine output
- **Intra-burst frequency:** 50 Hz
- **Pulses per burst:** 3
- **Burst Frequency:** 5 Hz
- **Burst duration:** 200 msec
- **Bursts per cycle:** 10 (2 seconds)
- **Pause within cycle:** 8 seconds
- **Cycle duration:** 10 seconds
- **Number of cycles:** 20
- **Total pulses per site:** 600
- **Total pulses per session:** 1800
